# Neuroimaging in Leber Hereditary Optic Neuropathy: State-of-the-art and future prospects

**DOI:** 10.1016/j.nicl.2022.103240

**Published:** 2022-10-25

**Authors:** Hugo T. Chow-Wing-Bom, Martina F. Callaghan, Junqing Wang, Shihui Wei, Frederic Dick, Patrick Yu-Wai-Man, Tessa M. Dekker

**Affiliations:** a Institute of Ophthalmology, University College London (UCL), London, United Kingdom; b Birkbeck/UCL Centre for NeuroImaging, London, United Kingdom; c Wellcome Centre for Human Neuroimaging, UCL Queen Square Institute of Neurology, University College London, London, United Kingdom; d Department of Ophthalmology, The Chinese People’s Liberation Army General Hospital, The Chinese People’s Liberation Army Medical School, Beijing, China; e Department of Psychological Sciences, Birkbeck, University of London, United Kingdom; f Department of Experimental Psychology, UCL, London, United Kingdom; g John van Geest Centre for Brain Repair and MRC Mitochondrial Biology Unit, Department of Clinical Neurosciences, University of Cambridge, Cambridge, United Kingdom; h Cambridge Eye Unit, Addenbrooke’s Hospital, Cambridge University Hospitals, Cambridge, United Kingdom; i Moorfields Eye Hospital NHS Foundation Trust, London, United Kingdom

**Keywords:** Optic neuropathies, LHON, MRI, DTI, Optic pathways

## Abstract

Leber Hereditary Optic Neuropathy (LHON) is an inherited mitochondrial retinal disease that causes the degeneration of retinal ganglion cells and leads to drastic loss of visual function. In the last decades, there has been a growing interest in using Magnetic Resonance Imaging (MRI) to better understand mechanisms of LHON beyond the retina. This is partially due to the emergence of gene-therapies for retinal diseases, and the accompanying expanded need for reliably quantifying and monitoring visual processing and treatment efficiency in patient populations. This paper aims to draw a current picture of key findings in this field so far, the challenges of using neuroimaging methods in patients with LHON, and important open questions that MRI can help address about LHON disease mechanisms and prognoses, including how downstream visual brain regions are affected by the disease and treatment and why, and how scope for neural plasticity in these pathways may limit or facilitate recovery.

## Highlights

• MRI reveals that LHON’s neural aetiology includes impacts beyond the retina.
• Effects are not limited to visual structures, but extend to non-visual regions.
• Why and when changes occur and link to function and other diseases remains unclear.
• New MRI methods (e.g., qMRI, g-ratio) may provide mechanistic insights into LHON.
• MRI could help inform and understand the effect of future therapies for LHON.

## 1 Introduction

Leber Hereditary Optic Neuropathy (LHON) is a primary mitochondrial DNA (mtDNA) disorder characterised by bilateral subacute loss of central vision (Yu-Wai-Man and Chinnery, 2000, Carelli et al., 2019). The pathological hallmark is the preferential loss of retinal ganglion cells (RGCs) within the inner retina, causing optic nerve degeneration and loss of vision. The prevalence of this mitochondrial optic neuropathy is ∼1 in 31,000 to 50,000 in the Northern European population (Yu-Wai-Man et al., 2003, Puomila et al., 2007). As the most common inherited optic neuropathy, LHON is an important cause of inherited blindness in young adults, with a higher prevalence in males, and a peak age of onset between the ages of 15–35 years (Yu-Wai-Man and Chinnery, 2000, Yu-Wai-Man et al., 2016, Carelli et al., 2019). Whilst promising regenerative therapies are currently being developed, idebenone is the only approved treatment to date for LHON. Idebenone stimulates ATP production by bypassing the complex I of the mitochondrial respiratory chain, which is impaired in LHON (Catarino and Klopstock, 2017), resulting in varying degrees of visual acuity improvements. Although most patients with LHON will develop isolated optic nerve involvement, a subgroup of patients manifest additional neurological features as part of a syndromic “LHON plus” phenotype, or features associated with multiple sclerosis-like illness. Given the major role that the central visual pathways play in visual function, disease progression beyond the retina may contribute substantially to the prognosis of LHON.

Given its flexibility and high spatial resolution, Magnetic Resonance Imaging (MRI) is a particularly valuable imaging modality for uncovering the effects of LHON on central visual pathways. In particular, MRI makes it possible to localise brain regions, structures, and processes linked to specific types of visual field loss. Recent structural and functional MRI studies of LHON have identified widespread pathological changes in different stages of the disease process, and across the visual system, from the optic tracts to the cortex (Barcella et al., 2010, Manners et al., 2015, Takemura et al., 2019, Long et al., 2019, Jonak et al., 2020b). These studies employ various informative MRI methodologies, which can answer important open questions about LHON. However, only a few of these methodologies are routinely used in clinics. As a result, techniques and approaches that could contribute to treatment and rehabilitation design may be overlooked. In this review, we discuss the new insights into the underlying pathophysiology that these studies provide, the important open questions that could be addressed using new MRI methods, and the challenges that need to be overcome to do so.

Specifically, we evaluate what is currently known from MRI research about the following questions about the neural mechanisms of LHON:

• Which changes in neural function and structure occur beyond the retinal ganglion cell layer in LHON, and when do they occur in time, i.e., when in the disease do changes in post-retinal brain structures from optic nerve to visually driven cortex occur?
• What are the mechanistic causes of these neural changes in LHON, and which microstructural processes might they reflect, i.e., demyelination and axonal loss?
• How do changes in neural structure and function predict visual outcome?
• What are the key challenges and limitations for current MRI research on LHON?
• What are the most important open questions about LHON that MRI could help resolve in the future?

By addressing these questions, we aim to provide a more comprehensive picture of the neural mechanisms of LHON than can be offered by RGC pathology in the retina alone. Given the key role post-retinal pathways play in visual function, these insights and methods may help inform prognoses and increase understanding of how, when, and why treatments work best. This is particularly important and timely in the light of new regenerative therapies (i.e., gene therapies; see response to Q5 in section 4) currently being developed for LHON, which would benefit from this knowledge. Other neuroimaging modalities such as electroencephalography (EEG) or positron emission tomography (PET) have also been used to study LHON, but go beyond the scope of this review.

### 1.1 Molecular genetics

LHON is primarily due to one of three pathogenic mtDNA mutations: m.11778G > A (most common), m.3460G > A, and m.14484 T > C. These affect the oxidative phosphorylation in the mitochondria of RGCs, preferentially within the papillomacular bundle, resulting in degeneration of RGCs and optic nerve axons. In a smaller proportion of cases where patients test negative to all common mutations, whole mitochondrial genome sequencing can help with the diagnosis of the disease (Moore & Yu-Wai-Man, 2021). Although LHON is commonly caused by point mutations in the mitochondrial DNA, patients with LHON-like phenotypes sometimes carry recessive nuclear genes (Moore and Yu-Wai-Man, 2021, Stenton et al., 2021). These diverse genetic aetiologies paint a more complex picture of LHON than was previously portrayed and highlight the challenges for diagnosis in some cases.

### 1.2 Clinical features

All types of RGCs (i.e., parvocellular, magnocellular and koniocellular cells) are impacted in LHON; however, parvocellular (P) cells within the papillomacular bundle are usually the most affected at early stages of the disease (i.e., acute phase), likely due to fewer mitochondria and smaller axons (Sadun et al., 2002, Majander et al., 2017). As a result of this, central vision rapidly deteriorates bilaterally, and colour vision and contrast sensitivity to high spatial frequencies are greatly diminished (Carelli et al., 2019). Optical Coherence Tomography has also revealed dynamic LHON-related changes of the retinal fibre nerve layer (RNFL), with initial thickening during the asymptomatic and early acute phases (particularly in the temporal and inferior quadrants), followed by a thinning of the RNFL as the disease progresses to the chronic phase (Savini et al., 2005, Barboni et al., 2005, Zhang et al., 2014). Other clinical characteristics of LHON include pallor and increased blood flow (i.e., hyperaemia) in the optic disc, and tortuosity of the central retinal vessels (Yu-Wai-Man and Chinnery, 2000, Sadun et al., 2002, Savini et al., 2005, Barboni et al., 2005, Zhang et al., 2014, Majander et al., 2017, Carelli et al., 2019). There is also evidence for oedema of the optic nerve during the acute phase (Frisén, 2017, Blanc et al., 2018).

Clinical observations of LHON progression have led to the description of four different stages (Carelli et al., 2019): 1) asymptomatic (LHON carriers with no clinically significant symptoms); 2) subacute (<6 months after clinical onset); 3) dynamic (6–12 months after clinical onset); and 4) chronic (>12 months after clinical onset). From here on, we will use the term *acute* to describe the period within the first year after clinical onset of the disease (i.e., encapsulating both subacute and dynamic phases), which is mainly characterised by the loss of RGCs.

In some cases, however, loss of vision is not definitive. A proportion of LHON patients (<20 %, all genotypes combined) experiences spontaneous visual recovery up to typical visual acuity levels a few years after disease onset (Carelli et al., 2019, Zuccarelli et al., 2020). A recent *meta*-analysis by Newman et al. (2020), which combined results from 12 prospective and 3 retrospective studies for a total of 695 LHON patients, all harbouring the m.G11778A variant, revealed that about 14 % of these patients presented a history of visual recovery, although some may be due to idebenone use. Most frequent recovery has been observed in those carrying the relatively rare m.T14484C variant (∼40–70 % of recovery cases; Carelli et al., 2019, Zuccarelli et al., 2020), raising the question of why spontaneous recovery is less frequent in patients with the G11778A variant compared to those with rarer forms of LHON. Visual recovery can also be achieved via the use of idebenone, which is the only approved treatment for LHON to date. This drug stimulates ATP production by bypassing the complex I of the mitochondrial respiratory chain which is impaired in LHON (Catarino and Klopstock, 2017), resulting in patients experiencing varying degrees of visual acuity improvements. Although idebenone has been shown to improve vision in a subset of LHON patients, recently emerging gene therapies for LHON show promise for offering a ‘one-shot’ treatment that could either stop progression or even regenerate functions. This increases the urgency of understanding neural plasticity in different stages of this disease.

### 1.3 LHON plus phenotype and comorbidities with other diseases

The majority of LHON patients develop symptoms limited to loss of foveal vision, colour vision, and acuity, with diagnosed pathology limited to the optic nerve and structures along the visual pathways. In rare cases, patients develop additional neurological features, such as lack of muscular control, tremors, and cardiac arrhythmia. Patients with this variant of the disease, described as *LHON Plus*, also present with demyelinating lesions in their central nervous system, which has led to associations between LHON and multiple sclerosis (Matthews et al., 2015, Yu-Wai-Man et al., 2016, Carelli et al., 2019). For instance, similarities in clinical presentation and the appearance and location of brain lesions have been reported in LHON-MS and MS patients (Pfeffer et al., 2014, Inglese et al., 2001, Matthews et al., 2015). Moreover, despite no clear relationship between LHON mtDNA variants and MS risk, an association between mtDNA haplogroups J, T, and JT, and MS risk has been reported in MS patients (Andalib et al., 2013, Bargiela and Chinnery, 2019 for reviews). This association is of interest as a potential link between haplogroups and visual failure in LHON patients has been observed in the literature (Hudson et al., 2007). This leads to speculation regarding whether a relationship exists between haplogroups in LHON-MS and MS patients, leading to similarities in clinical presentation and brain alterations.

The involvement of mitochondrial mutations in both MS and LHON raises questions on the impact of MS-related mitochondrial dysfunction on the pathology of LHON, and vice versa (Matthews et al., 2015, Bargiela and Chinnery, 2019, Rościszewska-Żukowska and Bartosik-Psujek, 2020). A fuller understanding of the relationship between LHON and demyelinating diseases could lead to potential insights into disease mechanisms, such as the role of mitochondrial dysfunction and demyelination. Finally, exploring the relationship between genotype and brain alterations might provide information for future prevention, monitoring, and treatment of these diseases.

## 2 Methods

### 2.1 Criteria for study selection

Currently, there is no systematic review or *meta*-analysis summarising pre-treatment MRI changes in the LHON literature. We intend to address this gap by providing a review of the reported structural and functional changes in the brains of patients affected by “pure” LHON, without treatment or other known neurological diseases. Our approach was as follows:

Using PubMed with keywords: LHON and MRI, 113 results were found. References of identified papers were also evaluated, resulting in a total of 120 studies. Studies were excluded if they were animal studies, published in a language other than English, did not use neuroimaging (e.g., surveys or clinical examinations without MRI) or used MRI modalities that were not the focus of this review (e.g., Magnetic Resonance Spectroscopy, see section 2.2. for included MRI methods). Additional exclusion criteria included studies solely assessing the effectiveness of a drug or treatment, or looking at LHON that was comorbid with other diseases which did not separately report changes or differences in patients with “pure” LHON. We also excluded case studies that involved child-onset (<15 years) disease, as LHON most commonly occurs in late adolescence or early adulthood.

This resulted in a total of thirty studies being selected for this review. Eight of these reported qualitative changes in the brain. The remaining twenty-two studies quantified neural differences between patients and healthy controls but covered a wide range of MRI modalities (e.g., structural MRI, DTI, qMRI). Within modality, the studies differed widely in terms of the regions of interest that were investigated (e.g., optic nerve, optic radiation, subcortical and cortical regions). This made it difficult to compare data from these studies quantitatively in any meaningful way, rendering a systematic review or *meta*-analysis unfeasible. Instead, we therefore systematically summarise and critically evaluate this literature. Table 1 lists the reviewed papers.

**Table 1:** List of studies about MRI changes in the LHON brain, reviewed in section 3.

QUANTITATIVE STUDIES	QUALITATIVE STUDIES
Barcella et al., 2010, *Human Brain Mapping*	Batioǧlu et al., 2003, *Journal of Neuro-Ophthalmology*
Blanc et al., 2018, *Journal of Neuro-Ophthalmology*	Honda et al., 2006, *Rinsho Shinkeigaku*
D'Almeida et al., 2013, *Neuroimage*	Lamirel et al., 2010, *Journal of Neurology, Neurosurgery, and Psychiatry*
Evangelisti et al., 2021, *Biochemical Pharmacology*	Mercuri et al., 2017, *Journal of Neuro-Ophthalmology*
Grochowski et al., 2020, *Journal of Clinical Medicine*	Ong et al., 2013, *Neurology*
Grochowski et al., 2021, *Journal of Clinical Medicine*	Phillips et al., 2003, *Archives of Ophthalmology*
Inglese et al., 2001, *Journal of Neurology, Neurosurgery, and Psychiatry*	Thouin et al., 2013, *PLoS ONE*
Jonak et al., 2020, *Brain Science*	Vaphiades et al., 2003, *Journal of Neuro-Ophthalmology*
Jonak et al., 2020, *Journal of Clinical Medicine*	
Jonak et al., 2021, *Neuroscience*	
Long et al., 2019, *Journal of Neurology*	
Manners et al., 2015, *American Journal of Neuroradiology*	
Mashima et al., 1998, *Journal of Neurology, Neurosurgery, and Psychiatry*	
Mateus et al., 2016, *Brain Structure and Function*	
Matthews et al., 2015, *Journal of Neurology, Neurosurgery, and Psychiatry*	
Milesi et al., 2012, *Journal of Neurology*	
Ogawa et al., 2014, *Investigative Ophthalmology and Visual Science*	
Rizzo et al., 2012, *PLoS ONE*	
Rocca et al., 2011, *PLoS ONE*	
Takemura et al., 2019, *Neuroimage Clinical*	
Wang et al., 2017, *European Journal of Radiology*	
Zhang et al., 2021, *Neuroimage Clinical*	

### 2.2 Neuroimaging methods used to study LHON

MRI is a non-invasive method that can be used to visualise the integrity of anatomical structures or evaluate dynamic metabolic function in the brain or other body areas (Symms et al., 2004). Some MRI applications, in particular those described under structural MRI, are widely used in clinics; for example, to visualise the shapes and sizes of different brain structures to assess atrophy in these regions. *Structural MRI* methods exploit the sensitivity to brain tissue microstructure that MRI properties, such as the longitudinal (T1) and transverse (T2) relaxation times. Lesions or potential inflammatory responses in the brains of LHON patients can alter these physical parameters and therefore the appearance of the different tissues in qualitative MRI images (e.g., brightness, size).

*Quantitative MRI* goes a step further by using (bio)physical signal models to quantify tissue properties, e.g., T1 and T2, directly in standardised units. This enhances the specificity of the measurement and therefore its capacity to characterise the microstructural integrity of the human brain in-vivo. This approach is valuable in enabling the direct comparison of measures between sites and within individuals over time, thereby allowing disease progression or therapeutic intervention to be monitored more objectively across sites or timepoints. In addition, a rich range of metrics (e.g., relaxation times, proton density, macromolecular volume fraction, magnetic susceptibility) can be quantified, with greater sensitivity to specific biological quantities such as myelin, iron, or water (Weiskopf et al., 2015, Weiskopf et al., 2021). Some measures, however, are only semi-quantitative: for instance, the *magnetisation transfer ratio (MTR)* quantifies the degree of signal reduction that occurs following the application of an “off-resonance” pulse that selectively saturates signal originating in macromolecules, such as myelin (Grossman et al., 1994, Henkelman et al., 2001), rather than offering a direct measure of macromolecule concentrations. More robust measures have recently been developed that account for the influence of spatial variability in the generation of magnetisation and in T1 times (Helms et al., 2008) on quantitative MRI measures.

*Diffusion imaging* has also been used to investigate white matter integrity across the LHON brain. *Diffusion tensor imaging* provides outcome measures such as *Mean Diffusivity (MD) and Fractional Anisotropy (FA)*, which summarise the ease and directionality of diffusion (e.g., equal in all directions, or preferentially along one specific direction) within a voxel respectively. Additional measures can also be obtained, such as the diffusivity along the 'principal axis' of a fibre (*Axial Diffusivity – AD*), or the average diffusivity along the two minor axes (*Radial Diffusivity – RD*). Although the brain’s microstructural features are not obtained directly from diffusion signal models, the derived model parameters can be used to infer features such as the long axes of larger neural white matter tracts. The consistency of changes or between-group differences in these parameters with processes like demyelination and axonal loss can also be assessed (Huisman, 2010, Wheeler-Kingshott and Cercignani, 2009, Winklewski et al., 2018). This is of particular interest for understanding LHON mechanisms since loss of both axons and myelin is thought to occur in this disease (Yu-Wai-Man et al., 2016).

Finally, *functional MRI (fMRI)* allows the study of dynamic functional changes in the brain, typically via the Blood-Oxygenation-Level-Dependent (BOLD) response (Buxton, 2013). This signal is sensitive to local changes in blood oxygenation over time following neural activity (Logothetis, 2002), and acts as a proxy for neural activation, either in response to specific stimuli (e.g., a visual stimulus), or functional connectivity as result of correlations between activity in remote brain regions under rest or other conditions. Using fMRI, it is possible to quantify loss or regain of sensitivity to retinal inputs in the brain, and assess how signals are transmitted across the hierarchy of visual cortex regions (Ritter et al., 2019; Farahbakhsh et al., 2021).

## 3 Structural and functional changes in the LHON brain

As LHON is a fast-progressing disease, allowing only a short time window for the recruitment of patients in the acute phase, most studies to date have primarily recruited patients well after their disease onset. Here, we therefore first focus on post-retinal changes reported in the brains of people with *chronic* LHON. We then review the few MRI studies on potential neural changes during *acute* LHON, as well as those in the brains of *asymptomatic* carriers of genetic mutations that cause LHON. Tables summarising findings can be found throughout this section.

### 3.1 Chronic LHON

#### 3.1.1 Pre-geniculate structural changes

**Fig. 1:**
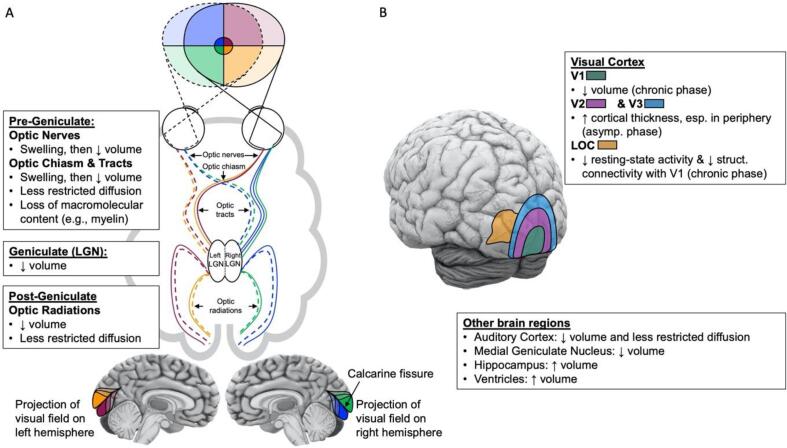
Summary of structural and functional changes A) along the visual pathways, and B) across the whole brain, in LHON.

Retinal ganglion cell axons project directly to the lateral geniculate nucleus (LGN; Fig. 1A). Axons originating from the nasal side of the retina (receiving light from the ipsilateral visual field) decussate to the opposite hemisphere at the optic chiasm, while axons originating from the temporal side of the retina (receiving light from the contralateral visual field) do not. As a result, information from the right visual field is processed in the left hemisphere and information from the left visual field in the right hemisphere. Before the optic chiasm, axonal projections from the retinal ganglion cells are termed *optic nerves;* after the partial decussation in the chiasm, they are termed *optic tracts*.

In line with the characteristic loss of RGCs in LHON, Inglese et al. (2001) reported reductions of optic nerve volume in 10 adults with chronic LHON and 4 adults with chronic LHON-MS, compared to age- and sex-matched normal-sighted controls (Table 2). The authors also found reduced MTR in the optic nerve of both patient groups compared to controls, with lower values in LHON-MS patients compared to those with chronic LHON. This suggests alterations in the white matter microstructure of the optic nerves in these patients. More recently, Wang et al. (2017) observed reduced fractional anisotropy, and increased mean, axial, and radial diffusivities in DTI measures from the optic nerves of 25 adults with chronic LHON compared to age-, and sex-matched, normal-sighted controls (Table 2). The authors also found that LHON patients with strong visual impairment (i.e., >3.0 logMAR) showed less well-constrained diffusion (i.e., fractional anisotropy values of <0.3) along the optic nerve, which in turn correlated with thinner peripapillary RNFL confirming that DTI measures of the optic nerve have functional relevance. These in-vivo MRI results parallel those of a post-mortem investigation showing severe axonal loss (99 %) in the optic nerve of a 75-year-old female LHON patient carrying the m.G3460C mutation who was first diagnosed with LHON at the age of 22 years (Rizzo et al., 2012).

**Table 2:** Structural changes observed in pre-geniculate visual regions of LHON individuals.

PRE-GENICULATE VISUAL REGIONS – STRUCTURAL CHANGES										
Reference	Normal-Sight. Controls (M ± SD age; [Range])	DISEASE STAGE			Mutation Type (N Individuals)	Notes	MRI Modalities	REGION OF INTEREST		Correlates
		Asymp. (M ± SD age; [Range])	Acute (M ± SD age; [Range])	Chronic (M ± SD age; [Range])				Optic Nerves	Optic Chiasm/Tracts	
Inglese et al., 2001	N = 20 (37.3 ± 12.3yrs) Age- and sex-matched			10 LHON (32.3 ± 12.3yrs) 4 LHON-MS (44.5 ± 8.5yrs) Disease: 1-26yrs (LHON); 2-21yrs (LHON-MS)	G11778A (11) G3460A (2) T14484C (1)	Idebenone (2 LHON, 2 LHON-MS)	Struct. MRI MTI	↓ volume ↓ MTR		Optic nerve volume with disease duration (r = -0.7, p = 0.005)
Rizzo et al., 2012	N = 22 (37 ± 17yrs) Age- and sex-matched 1 post-mort. (♀75yrs)	N = 11 (45 ± 15yrs) Normal-sight.		22 LHON (33 ± 11yrs) Disease: 1–35yrs 1 post-mort. LHON (♀75yrs) Disease: 53yrs	G11778A (22: 8 asymp.) G3460A (10: 3 asymp.) T14484C (1 LHON) Post-mort.: G3460A	Idebenone(22 LHON) VA recovery (3 LHON)	Struct. MRI DWI Cell. Imag.	**Post-Mortem:**Severe axonal loss (↓99 %)		
Wang et al., 2017	N = 28 (27.8 ± 10.1yrs; [18-44yrs]) Age- and sex-matched			25 LHON (28.08 ± 10.66yrs; [18-50yrs]) Disease: 1.1–35.2yrs	G11778A (9) G3460A (2) T14484C (4)		Struct. MRI DTI	↓ FA ↑ MD, AD, and RD		FA in optic nerve with VA (r = -0.404, P = 0.003), mean defect of visual field (r = -0.445, P = 0.001) & average pRNFL thickness (r = 0.321, P = 0.023)
Barcella et al., 2010	N = 12 (M = 33.2yrs; [23-58yrs]) Age- and sex-matched			12 LHON (M = 33.6yrs; [20-60yrs]) Disease: 1-23yrs	G11778A (5) G3460A (4) T14484C (3)		Struct. MRI		↓ volume	
Jonak et al., 2020b	N = 15 (32.5 ± 7.4yrs) Age- and sex-matched			15 LHON (36.21 ± 14.41yrs) Disease: 1-40yrs	G11778A (15)		Struct. MRI		↓ volume	
Milesi et al., 2012	N = 25 (M = 35yrs; [25-57yrs]) Age-matched			13 LHON (M = 36.0yrs; [21-61yrs]) Disease: 2-34yrs	G11778A (8) G3460A (3) T14484C (2)		Struct. MRI DTI		↓ FA	FA in optic tracts with VA (r = 0.57, p = 0.04)
Ogawa et al., 2014	N = 14 (32.1 ± 5.4yrs; [24-40yrs]) Age-matched			6 LHON (37.5 ± 12.4yrs; [22-59yrs]) Disease: 1-22yrs	G11778A (6)		Struct. MRI DWI		↓ FA, esp. in portions close to LGN ↓ AD, slight ↑ RD	
Takemura et al., 2019	N = 20 (29.7 ± 9.7yrs; [19-44yrs]) Age-matched			7 LHON (28.6 ± 9.3yrs; [19-44yrs]) Disease: 1-22yrs	G11778A (5) T14484C (2)	Idebenone (3) VA recovery (1)	Struct. MRI DTI qMRI		↓ FA ↑ qT1	
Batioǧlu et al., 2003			1 Case-study: ♂30yrs Disease < 1 yr		G11778A		Struct. MRI		T2 hyperintensity	
Honda et al., 2006			1 Case-study: ♂46yrs Disease < 1 yr		G11778A		Struct. MRI		T2 hyperintensity & no contrast enhancement	
Lamirel et al., 2012			1 Case-study: ♀19yrs 10 months post-onset		G11778A		Struct. MRI	Left T2 hyperintensity & pre-chiasmal contrast enhancement gone 1 month later	T2 hyperintensity & enlargement on the left side	
Blanc et al., 2018			N = 28 (M = 38.3yrs) Disease < 1 yr		G11778A (21) G3460A (4) T14484C (2) 14487 (1 – rare)		Struct. MRI	**3/28 patients (11 %):** T2 hyperintensity of the canalicular and intracranial segments & no contrast enhancement	**16/28 patients (57 %):** – Optic nerve T2 hyperintensity extend to the chiasm - Qualitative enlargement & no contrast enhancement	
Phillips et al., 2003			2 Case-studies: ♂7yrs & ♂19yrs Disease < 1 yr		G11778A (1) G3460A (1)		Struct. MRI	**♂7yrs:** Enlargement and intracranial optic nerve contrast enhancement, seen 2 months post-onset, reduced after 5 months and gone after 2yrs **♂19yrs:** No contrast enhancement from 1 to 4 months post-onset	**♂7yrs:** Enlargement & no contrast enhancement, seen ca. 2 months post-onset but not after 2yrs. **♂19yrs:** Enlargement & no contrast enhancement, seen ca. 1 month post-onset and still present after 4 months.	
Ong et al., 2013			1 Case-study: ♂19yrs Disease < 1 yr		G11778A		Struct. MRI		Asymmetrical T2 hyperintensity, enlargement & contrast enhancement	
Mercuri et al., 2017			1 Case-study: ♀30yrs 4 months post-onset		T14484C T3394C		Struct. MRI	Enlargement and contrast enhancement	Enlargement & contrast enhancement	
Vaphiades et al., 2003			3 Case-studies: ♂7yrs, ♂18yrs & ♂24yrs Disease < 1 yr		G11778A (2) G3460A (2)		Struct. MRI	Bilateral contrast enhancement in all patients	Enlargement, only in ♂7yrs	
Mashima et al., 1998			N = 5 (M = 25.8yrs; [21-38yrs]) Disease < 1 yr		G11778A (5)		Struct. MRI	Bilateral hyperintensity toward the orbital apex, ca. 6–12 months post-onset		
Thouin et al., 2013			3 Case-studies: ♂58yrs, ♂63yrs & ♂72yrs Disease < 1 yr		G11778A (3)		Struct. MRI	Normal & no contrast enhancement for all patients	Normal & no contrast enhancement for all patients	

MTI: magnetisation transfer imaging; MTR: magnetisation transfer ratio; DTI: Diffusion Tensor Imaging; DWI: Diffusion Weighted Imaging; qMRI: quantitative MRI; FA: fractional anisotropy; MD: mean diffusivity; AD: axial diffusivity; RD: radial diffusivity: RNFL: retinal nerve fibre layer; pRNFL: peripapillary RNFL; VA: visual acuity.

Taken together, these studies suggest that changes in optic nerve properties in LHON may reflect myelin loss and axonal death. However, the time course of these changes, and which factors contribute to them, is less clear: while Inglese et al. (2001) found that overall loss of optic nerve volume in adults with chronic LHON correlated with disease duration ranging from 1 to 26 years, Wang et al. (2017) found that none of the DTI parameters correlated with age or disease duration in their cohort. Given these conflicting findings, it is therefore still unclear whether degeneration of optic nerve integrity principally occurs during the acute stage of LHON, or whether degeneration is more prolonged.

Beyond the optic nerves, reductions of volume have also been reported in the optic chiasm and tracts of adults with chronic LHON, compared to age-matched normal-sighted controls (Barcella et al., 2010, Jonak et al., 2020b; Table 2). None of these studies found correlations of volume with disease duration, visual acuity, or visual field defects. When compared to age-match normal-sighted controls, reduced FA was also found in the optic tracts of adults with chronic LHON (Milesi et al., 2012, Ogawa et al., 2014, Takemura et al., 2019), which paired with a reduction in visual acuity (Milesi et al., 2012) supporting the functional relevance of DTI measures. Using quantitative MRI, Takemura et al. (2019) showed reduced FA and increased qT1 values in the optic tracts of 7 adults with chronic LHON, compared to 20 adult normal-sighted controls. This is further evidence that loss of macromolecular content (i.e., from axonal or myelin loss) is pervasive across the retinogeniculate pathway.

In sum, while there is clear evidence for tissue loss with functional relevance across the pre-geniculate visual pathway in chronic LHON (Table 2; Fig. 1A), it is still unclear which microcellular tissues are affected when, and whether most damage occurs early in the disease or whether gradual progression also plays an important role. Addressing these questions will be crucial for optimising timing of treatment regimes.

#### 3.1.2 Geniculate and post-geniculate structural changes in optic radiation and primary visual cortex

Visual information is projected directly from the retina onto the parvo- and magnocellular layers of the LGN, the thalamic relay station of the visual system. It is subsequently conveyed to the primary visual cortex (V1) via single synapse projections termed the optic radiations (Fig. 1A). Whereas alterations of pre-geniculate structures – such as loss of cells in the retinal nerve fibre layer (RNFL) and altered optic nerve integrity – are primary symptoms of LHON, several open questions can be raised regarding these ‘downstream’ structures, including (i) whether, how, and when LHON affects these regions, and (ii) whether such changes might be driven by visual deprivation due to retinal degeneration, or by primary disease processes in post-geniculate tracts. Answering these questions will be crucial for understanding the time course and mechanisms of potentially treatment-hindering central neural atrophy in LHON.

In the aforementioned post-mortem study on LHON (in a 75-year-old patient), Rizzo et al. (2012) found that the severe axonal loss described in the optic nerve (see above) was accompanied by atrophy of neuronal soma and loss of neuron density in both M- and P-cell layers of the LGN. In line with this, MRI studies have detected reductions in volume in the LGN, optic radiations, and primary visual cortex (V1) of adults with chronic LHON, compared to normal-sighted adult controls (Barcella et al., 2010, Jonak et al., 2020b, Jonak et al., 2020a; Table 3; Fig. 1A). Barcella et al. (2010) found that loss of optic radiation and V1 volume correlated with decreased average and temporal peripapillary RNFL thickness in 12 adults with chronic LHON, but not with visual acuity or visual field sensitivity.

**Table 3:** Structural changes observed in geniculate and post-geniculate visual regions of LHON individuals.

GENICULATE AND POST-GENICULATE VISUAL REGIONS – STRUCTURAL CHANGES										
Reference	Normal-Sight. Controls (M ± SD age; [Range])	DISEASE STAGE			Mutation Type (N Individuals)	Notes	MRI Modalities	REGION OF INTEREST		Correlates
		Asymp. (M ± SD age; [Range])	Acute	Chronic LHON (M ± SD age; [Range])				LGN	Optic Radiations	
Rizzo et al., 2012	N = 22 (37 ± 17yrs) Age- and sex-matched 1 post-mort. (♀75yrs)	N = 11 (45 ± 15yrs) Normal-sight.		N = 22 (33 ± 11yrs) Disease: 1–35yrs 1 post-mort. (♀75yrs) Disease: 53yrs	G11778A (22: 8 asymp.) G3460A (10: 3 asymp.) T14484C (1 LHON) Post-mort.: G3460A	Idebenone(22 LHON) VA recovery (3 LHON)	Struct. MRI DWI Cell. Imag.	**Post-mortem:** – ↓ mean soma size in all 6 layers (layers 1–2: ↓ 41.6 %, layers 3–6: ↓ 44.7 %) – ↓ mean neuron density in all 6 layers (layers 1–2: ↓ 28.5 %, layers 3–6: ↓ 28.7 %)	↑ MD in LHON compared to asymp. & controls No change in MD between asymp. & controls	↑ MD in optic radiations of chronic LHON associated with lack of visual recovery (B = 0.060; P < 0.01) and disease duration (B = 0.002;P < 0.05)
Barcella et al., 2010	N = 12 (M = 33.2yrs; [23-58yrs]) Age- and sex-matched			N = 12 (M = 33.6yrs; [20-60yrs]) Disease: 1-23yrs	G11778A (5) G3460A (4) T14484C (3)		Struct. MRI		↓ volume	Right optic radiation volume with average pRNFL (r = 0.78, P_uncorr_ < 0.001)Left optic radiation volume with average pRNFL (r = 0.84, P_uncorr_ < 0.001) & temporal RNFL thickness (r = 0.79, P_uncorr_ < 0.001)
Jonak, Krukow, Jonak, et al., 2020	N = 15 (32.53 ± 7.42yrs) Age- and sex-matched			N = 15 (36.21 ± 14.41yrs) Disease: 1-40yrs	G11778A (15)		Struct. MRI	↓ volume of right LGN		Right LGN volume with right RNFL thickness (r = 0.891,p < 0.0001) & right optic nerve volume in its most proximal portion (r = 0.727,p = 0.001)
Ogawa et al., 2014	N = 14 (32.1 ± 5.4yrs; [24-40yrs]) Age-matched			N = 6 (37.5 ± 12.4yrs; [22-59yrs]) Disease: 1-22yrs	G11778A (6)		Struct. MRI DWI		↓ FA, esp. in subcomponents carrying lower and foveal inputs ↑ RD, slight ↓ AD	
Takemura et al., 2019	N = 20 (29.7 ± 9.7yrs; [19-44yrs]) Age-matched			N = 7 (28.6 ± 9.3yrs; [19-44yrs]) Disease: 1-22yrs	G11778A (5) T14484C (2)	Idebenone (3) VA recovery (1)	Struct. MRI DTI qMRI		↓ FA, esp. in subcomponents carrying foveal inputs No change in qT1 recovery	
Manners et al., 2015	N = 19 (37 ± 10yrs) Age- and sex-matched			N = 17 (37 ± 10yrs; [25-55yrs]) Disease: 1-43yrs	G11778A (13) G3460A (3) T14484C (1)	Idebenone (11) VA recovery (4)	Struct. MRI DTI		↓ FA and ↑ MD ↑ number of affected voxels if idebenone as a covariate (by 45 % for FA, 175 % for MD)	
Milesi et al., 2012	N = 25 (M = 35yrs; [25-57yrs]) Age-matched			N = 13 (M = 36.0yrs; [21-61yrs]) Disease: 2-34yrs	G11778A (8) G3460A (3) T14484C (2)		Struct. MRI DTI		↓ FA, ↑ MD and RD	

LGN: lateral geniculate nucleus; FA: fractional anisotropy; MD: mean diffusivity; RD: radial diffusivity; AD: axial diffusivity; RNFL: retinal nerve fibre layer; pRFNL: peripapillary RNFL; qT1: quantitative. T1.

DTI studies also reported altered diffusivity in post-geniculate tracts, with decreased FA and AD, and increased MD and RD in the optic radiations of adults with chronic LHON (Rizzo et al., 2012, Ogawa et al., 2014, Manners et al., 2015, Takemura et al., 2019; Table 3; Fig. 1A). These changes indicate less restricted diffusion along the principal direction of the diffusion tensor in the optic radiation white-matter tracts. Again, whether this is due to loss of axonal fibres, loss of myelinated membranes, and/or localised inflammation, cannot be distinguished based on these measures, but is important for understanding potential recovery mechanisms. Interestingly, when dividing optic radiations into subcomponents that convey visual inputs from different parts of the visual field, Ogawa et al., 2014, Takemura et al., 2019 both found reduced FA in the subcomponents that carry inputs from the fovea and lower visual field. Although these results need to be reproduced in a larger sample of patients, the location of the FA reduction is in line with the pattern of RGC loss across the retinal sheet, and particularly the preferential loss of P-cells leading to impaired central vision characteristic of LHON (Sadun et al., 2002, Majander et al., 2017). This suggests that post-geniculate degeneration may primarily be a knock-on effect of neuronal loss in the retina, rather than a shared disease process simultaneously affecting all these brain regions.

It is important to note that although less restricted diffusion is observed in both optic tract and optic radiation in LHON, the biological reason for this may differ. Two studies from the same group reported reduced axial diffusion in optic tracts and increased radial diffusion in the optic radiations (Ogawa et al., 2014, Takemura et al., 2019). Whilst reductions in axial diffusivity have been linked to axonal loss, reduced radial diffusivity is thought to reflect myelin integrity (Wheeler-Kingshott and Cercignani, 2009, Winklewski et al., 2018). However, inflammation along the visual pathway can also affect diffusivity (Winklewski et al., 2018). It is therefore possible that less restricted diffusion measured in chronic LHON involves loss of axons and inflammation in optic tracts, but loss of myelin in optic radiations.

Do changes in post-geniculate visual structures during the chronic phase occur suddenly or gradually? Whilst LGN and V1 volume correlated with RNFL thickness, these volume measures did not vary with disease duration (Barcella et al., 2010, Jonak et al., 2020b, Jonak et al., 2020a; Table 3). Moreover, no correlation was found between DTI measures in the optic tracts and those in the optic radiations (Takemura et al., 2019). The absence of a clear link between disease duration and post-geniculate changes of neural integrity might suggest that these changes mainly occur during the acute phase in tandem with or very soon after RGC loss. However, as mentioned above, there have been conflicting reports about whether structural changes correlate with disease length in LHON (Rizzo et al., 2012, Milesi et al., 2012, Manners et al., 2015). Interpreting these discrepancies is complicated by the typically small cohorts in studies on LHON, as well as whether patients received treatment with idebenone (Rizzo et al., 2012: N = 22/22 chronic patients; Manners et al., 2015: N = 11/17 chronic patients; Milesi et al., 2012: not reported). It is therefore still unclear when and why the posterior pre-cortical visual pathway is affected by LHON.

In sum, MRI studies have revealed structural differences, including reduced volume and impaired microstructural white-matter integrity, in the post-geniculate structures of adults with chronic LHON relative to controls (Table 3; Fig. 1A). Correlations between decreased RNFL thickness, field loss, and reduced volume and diffusion along multi-synaptic projections of the pre-cortical visual pathway, suggest that atrophies in post-geniculate neuronal tracts are in part linked to the loss of specific RGCs. However, correlations between disease duration, visual function, and neural changes in post-geniculate structures are inconsistent across studies. Therefore, the degree to which these result from visual deprivation or other pathological processes, and how and when these changes emerge and contribute to function as the disease progresses, is still debated. Moreover, it is unclear whether these changes reflect myelin and/or axonal loss, as current measures can only index these processes indirectly.

#### 3.1.3 Structural changes beyond the primary visual cortex

While most white matter abnormalities have been reported along the visual pathways in LHON, more widespread white matter changes have also been found (Table 4; Fig. 1B). This raises the question of more general neural pathological mechanisms in LHON beyond the ophthalmic regions. Rocca et al. (2011) used a tractography seed-clustering analysis to assess structural connectivity between the primary visual and auditory cortices, and the rest of the brain, in 13 adults with chronic LHON compared to 13 age- and sex-matched controls. Similar seed clusters emerged from this analysis run separately in the two groups, although the authors noted some qualitative differences. These include altered clustering from visual cortex seeds (e.g., in lateral occipital cortex, fusiform gyrus, temporal pole, and frontal pole) as well as from auditory cortex seeds (in a range of visual-, motor-, multimodal-, and sub-cortical regions), in line with widespread effects of chronic LHON on brain structure beyond V1 (Table 4). Future studies should confirm if these reflect systematic differences between patients with LHON and controls or random variation in the measure. In a recent DTI study involving 19 acute and 34 chronic adults with LHON, Zhang et al. (2021) showed that although highly connected hub structures across the brain seemed to be preserved, impaired structural network connectivity (reduced FA, increased RD) and abnormal connections were found between regions involved in peripheral processing of visual/auditory sensation and motor control such as dorsal and ventral visual, auditory, and basal ganglia areas (Table 4). Jonak et al. (2021) also showed altered structural connectivity using DTI, with fewer connections between brain regions of chronic patients, including the optic chiasm (as expressed in decreased degree value and betweenness centrality). This resulted in a shift of large-scale network topology that correlated with disease duration and suggests less centralised organisation, and potentially, less efficient transfer of information between brain regions.

**Table 4:** Structural changes observed in V1, and beyond V1 of LHON individuals.

V1 AND OTHER SUBCORTICAL AND CORTICAL REGIONS – STRUCTURAL CHANGES											
Reference	Normal-Sight. Controls (M ± SD age; [Range])	DISEASE STAGE			Mutation Type (N Individuals)	Notes	MRI Modalities	REGION OF INTEREST			Correlates
		Asymp. (M ± SD age; [Range])	Acute	Chronic LHON (M ± SD age; [Range])				V1	Extrastriate Cortex	Other Cortical Or Subcortical Regions	
Barcella et al., 2010	N = 12 (M = 33.2yrs; [23-58yrs]) Age- and sex-matched			N = 12 (M = 33.6yrs; [20-60yrs]) Disease: 1-23yrs	G11778A (5) G3460A (4) T14484C (3)		Struct. MRI	↓ V1 volume			Right V1 volume with: - Average pRNFL (r = 0.89, P_uncorr_ < 0.001)- Temporal RNFL thickness (r = 0.76, P_uncorr_ < 0.001) Left V1 volume with:- Temporal RNFL thickness (r = 0.89,P_uncorr_ < 0.001)
Rocca et al., 2011	N = 13 (M = 35.2yrs; [19-59yrs]) Age- and sex-matched			N = 13 (M = 35.6yrs; [20-61yrs]) Disease: 2-34yrs	G11778A (8) G3460A (3) T14484C (2)		Struct. MRI RS-fMRI DTI	**Structural connectivity:** **Between right V1 &** **-** TFC (LHON only), OFC (↑ vs controls), LOC & OFG (↓ vs controls) **Between left V1 &** **-** MTG & IT (LHON only), Frontal pole & LOC (↑ vs controls), Temporal pole (↓ vs controls)		**Structural connectivity:** **Between right auditory cortex &** - Frontal pole, pallidum, and SMG (LHON only) **Between left auditory cortex &** - LOC (LHON only) - MTG and IT (Controls only)	
Manners et al., 2015	N = 19 (37 ± 10yrs) Age- and sex-matched			N = 17 (37 ± 10yrs; [25-55yrs]) Disease: 1-43yrs	G11778A (13) G3460A (3) T14484C (1)	Idebenone (11) VA recovery (4)	Struct. MRI DTI			↓ FA and ↑ MD: auditory radiations, right superior corona radiata, SLF, and medial corpus callosum If treated with idebenone: lower MD values within anterior cingulum, genu of corpus callosum, olfactory tracts, and left prefrontal white-matter	
Jonak et al., 2020b	N = 15 (32.5 ± 7.4yrs) Age- and sex-matched			N = 15 (36.2 ± 14.4yrs) Disease: 1-40yrs	G11778A (15)		Struct. MRI			**↓ volume:** palladium and accumbens area **↑ volume:** lateral ventricles, temporal horns of lateral ventricles, 3rd & 4th ventricles	Volume of left and right lateral ventricles with disease duration (left: R = 0.656,p = 0.002; right: R = 0.755,p = 0.001) & age (left: R = 0.656,p = 0.007; right: R = 0.691, p = 0.004)
Jonak et al., 2020a	Same as above			Same as above	Same as above		Struct. MRI			↓ volume of MGN	
Grochowski et al., 2021	N = 15 (33.1 ± 7.2yrs) Age- and sex-matched			N = 15 (36.2 ± 14.9yrs) Disease: 1-41yrs	G11778A (15)		Struct. MRI			**↑ volume:** hippocampal fissure, hippocampal tail and body, subiculum body, all CA body, molecular layer HP body, GC-ML-DG body, whole hippocampal volume **↓ volume:** right fimbria	Volume of hippocampal fissure with disease duration (R = 0.675,p = 0.005) Volume of fimbria with disease duration (R = -0.595,p = 0.018)
Rizzo et al., 2012	N = 22 (37 ± 17yrs) Age- and sex-matched 1 post-mort. (♀75yrs)	N = 11 (45 ± 15yrs) Normal-sight.		N = 22 (33 ± 11yrs) Disease: 1–35yrs 1 post-mort. (♀75yrs) Disease: 53yrs	G11778A (22: 8 asymp.) G3460A (10: 3 asymp.) T14484C (1 LHON) Post-mort.: G3460A	Idebenone(22 LHON) VA recovery (3 LHON)	Struct. MRI DWI Cell. Imag.			**Between groups:** No difference in MD of prefrontal and cerebellar white-matter	
D’Almeida et al., 2013	N = 15 (26.2 ± 11.5yrs; [7-44yrs]) Age-matched	N = 15 (29.3 ± 13.5yrs; [8-47yrs]) Normal-sight.			G11778A (15)		Struct. MRI Retino. fMRI	**Between asymp. & controls:** No difference in V1 cortical thickness	**Between asymp. & controls:** - ↑ V2 cortical thickness, if age ≤ 21yo (N = 7 asymp.) - ↑ V3 cortical thickness, if age > 21yo (N = 8 asymp.)	**Between asymp. & controls:** No difference in cortical thickness of pre- and post-central gyri	Outer-macular RNFL thickness with:- V2 cortical thickness (r = 0.582,p = 0.023) - V3 cortical thickness (r = 0.537,p = 0.039)Age with (for N = 7 asymp. aged ≤ 21yo only):- V2 cortical thickness (r = -0.857, p < 0.001)- V3 cortical thickness (r = -0.833, p = 0.001)
Mateus et al., 2016	N = 24 (31.3 ± 13.5yrs; [7-54yrs]) Age-matched	Same as above			Same as above		Struct. MRI Retino. fMRI		↑ V2 and V3 cortical thickness, esp. in peripheral regions		Outer-macular RNFL thickness with: - V2 cortical thickness (r = 0.712,p = 0.0075)- V3 cortical thickness (r = 0.706,p = 0.0083)
Long et al., 2019	N = 15 (31.9 ± 10.2yrs; [11-44yrs]) Age and sex-matched	N = 14 (37.1 ± 12.7yrs; [9-52yrs]) Normal-sight.			G11778A (8) G3460A (2) T14484C (4)		Struct. MRI DTI			**AD**: no difference **↓ FA**: bilat. anterior thalamic radiations, bilat. corticospinal tracts, major and minor forceps, bilat. IFOF and left SLF **↑ MD**: bilat. anterior thalamic radiations, bilat. corticospinal tracts, minor forceps, bilat. IFOF, bilat. ILF, left SLF and bilat. uncinate fasciculi **↑ RD:** bilat. anterior thalamic radiations, bilat. corticospinal tracts, major and minor forceps, bilat. IFOF, bilat. ILF, bilat. SLF, and bilat. uncinate fasciculi	
Zhang et al., 2021	N = 36 ([9-44yrs]) Age and sex-matched		N = 19 (21.4 ± 11.6yrs; [10-57yrs]) Disease: 0.27 ± 0.22yrs	N = 34 (26.9 ± 11.7yrs; [13-53yrs]) Disease: 9.5 ± 10.6yrs	G11778A (41: 14 acute)T14484C (9: 5 acute)G3460A (3 chronic)		Struct. MRI DTI			Preserved rich-club organization **↓ FA, ↑ RD:** esp. in non-rich club components Abnormal feeder connections within dorsal visual areas, and between basal ganglia & dorsal visual area. Abnormal local connections between auditory cortex & dorsal and ventral visual area, between basal ganglia & ventral visual area	

MGN: medial geniculate nucleus; LOC: lateral occipital cortex; OFC: occipital fusiform cortex; OFG: occipital fusiform gyrus; TFC: temporal fusiform cortex; RNFL: retinal nerve fibre layer; pRNFL: peripapillary RNFL; DWI: Diffusion-weighted imaging; DTI: Diffusion-Tensor imaging; FA: fractional anisotropy; MD: mean diffusivity; RD: radial diffusivity; AD: axial diffusivity; CA: Cornu Ammonis; MTG: middle temporal gyrus; IT: inferior temporal gyrus; SMG: supramarginal gyrus; SLF: superior longitudinal fasciculus; ILF: inferior longitudinal fasciculi; IFOF: inferior fronto-occipital fasciculi; bilat.: bilateral.

In line with these data suggesting that neural symptoms of LHON may be widespread and beyond brain structures involved in vision, Manners et al. (2015) found reduced FA and smaller acoustic radiations in 17 adults with chronic LHON compared to 19 controls. Jonak et al. also reported reduced volume in the medial geniculate nucleus of 15 patients with chronic LHON compared to age- and sex-matched controls (Jonak et al., 2020a; Table 4). This subcortical region is located between the inferior colliculus and the auditory cortex and acts as the auditory thalamic relay. These observations are in line with hearing abnormalities sometimes reported in LHON patients (Rance et al., 2012). Further changes in subcortical structures have also been reported in chronic LHON, such as enlargement of the hippocampus (Grochowski et al., 2021) and ventricles (Jonak et al., 2020b), which correlated with disease duration as well as age (Table 4). Ventricle enlargement and increased cerebrospinal fluid volume are reliable morphometric features of neural atrophy. Collectively, this work suggests that LHON may have a broadly neurodegenerative nature, involving brain regions beyond the visual system (Fig. 1B).

#### 3.1.4 Functional changes

Connectivity across two brain regions can be assessed by quantifying how well neural signal fluctuations, as expressed in the functional MRI BOLD signal in these regions, are temporally coupled with each other (i.e., *functional connectivity*). By identifying regions with strongly correlated BOLD response time courses, it is possible to identify *networks* of brain regions that are presumably strongly co-activated during perception and cognition and therefore ‘wire together’ into functional units. These correlations can even be present during rest, i.e., resting state networks (Rosazza and Minati, 2011, Smitha et al., 2017, Seitzman et al., 2019).

Using resting-state fMRI, Rocca et al. (2011) found reduced spatiotemporal coupling in secondary visual networks of 13 adults with chronic LHON compared to 13 age- and sex-matched controls, more specifically in the right lateral occipital cortex and the right temporal occipital fusiform cortex (Table 5; Fig. 1B). This reduction correlated with disease duration and temporal RNFL and was paired with qualitatively reduced structural connectivity between right V1 and right lateral occipital cortex, as measured by DTI. These results suggest that extensive central retinal damage and loss of structural integrity along the visual pathways in chronic LHON, may be accompanied by reduced functional and structural connectivity between V1 and higher-order visual regions.

**Table 5:** Functional changes observed in V1, and beyond V1 of LHON individuals.

V1 AND OTHER SUBCORTICAL AND CORTICAL REGIONS – FUNCTIONAL CHANGES											
Reference	Controls (M ± SD age; [Range])	DISEASE STAGE			Mutation Type (N Individuals)	Notes	MRI Modalities	REGION OF INTEREST			Correlates
		Asymp. (M ± SD age; [Range])	Acute	Chronic (M ± SD age; [Range])							
								V1	Extrastriate Cortex	Other Cortical Or Subcortical Regions	
Rocca et al., 2011	13 normal-sight. (M = 35.2yrs; [19-59yrs]) Age- and sex-matched			13 LHON (M = 35.6yrs; [20-61yrs]) Disease: 2-34yrs	G11778A (8) G3460A (3) T14484C (2)		Struct. MRI RS-fMRI DTI	**Primary visual network of chronic LHON:** ↑ resting-state fluctuations in left cuneal cortex & right supracalcarine cortex	**Secondary visual networks of chronic LHON:** – ↑ resting-state fluctuations in left OFG & bilat. occipital poles - ↓ resting-state fluctuations in temp. OFC, corresponding with ↑ struct. connectivity between right V1 and right temp. OFC - ↓ resting-state fluctuations in right LOC, corresponding with ↓ struct. connectivity between right V1 and right LOC	**↑ resting-state fluctuations in chronic LHON:** - Right STG (incl. primary auditory cortex) - SMG, corresponding with ↑ struct. connectivity between right auditory cortex and right SMG	Disease duration with resting-state activity- Left cuneal cortex (r = 0.87,p = 0.003)- Right occipital pole (r = 0.87,p = 0.003) - Right LOC (r = -0.77,p = 0.01)- Right STG (r = 0.83,p = 0.02) Average RNFL thickness with resting-state activity- Left cuneal cortex (r = 0.88,p = 0.002) - Right STG (r = 0.79,p = 0.05)

LOC: lateral occipital cortex; OFC: occipital fusiform cortex; OFG: occipital fusiform gyrus; TFC: temporal fusiform cortex; SMG: supramarginal gyrus; STG: superior temporal gyrus; bilat.: bilateral; temp.: temporal.

In contrast, Rocca et al. (2011) also found *increased* spatiotemporal coupling in primary and secondary visual networks and in various non-visual networks, accompanied by increased structural connectivity in DTI in these regions (Table 5). These changes may reflect potential compensatory responses that may involve cross-modal plasticity (Rocca et al., 2011, Manners et al., 2015). However, whether changes in non-visual areas are caused by LHON or because of blindness is still unclear.

To date, no MRI study has directly investigated changes in visual function (e.g., visually evoked BOLD responses) in LHON patients. Yet, MRI studies in other retinal diseases suggest that changes in visual function may have important applications for monitoring functional change after treatment (see response to Q5 in section 4). Adapting and combining existing functional MRI paradigms and modelling approaches to study functional changes in LHON could shed light on potential links between cortical and behavioural changes in vision.

#### 3.1.5 Recovery after pharmacological therapy

Although there is no cure for LHON yet, idebenone has been proven safe to be used for the treatment of LHON and has shown positive outcomes for improving vision in LHON patients (Catarino and Klopstock, 2017). Several studies have reported bilateral or unilateral recovery of visual acuity and visual field sensitivity in small subsets of patients, with some even recovering a visual acuity of 1.0 in decimal units (i.e., “20/20 vision”) on the Snellen chart (Rizzo et al., 2012, Manners et al., 2015, Catarino and Klopstock, 2017, Pemp et al., 2021). However, recovery is far from ubiquitous in LHON patients treated with idebenone, and depends on several factors such as mutation, age of onset, disease duration, presence of other diseases such as multiple sclerosis, and duration of treatment. Moreover, beneficial effects can take as long as 30 months to emerge (Carelli et al., 2019). Evidence of improvements in brain measures have also been reported in LHON patients treated with idebenone using MRI. Greater volume and diameter of the optic nerves have been found in treated patients compared to untreated patients (Grochowski et al., 2020: N = 6 treated and N = 9 untreated patients), specifically in the part close to the optic chiasm. Lower mean diffusivity values have also been reported in optic radiations and non-visual regions of treated compared to untreated patients, including in the anterior cingulum, genu of corpus callosum, olfactory tracts bilaterally, and the left prefrontal WM, as measured in DTI (Manners et al., 2015: N = 11 treated and 6 untreated patients). Additionally, Rizzo et al. (2012) reported that structural integrity measures of the optic radiations could help discriminate between LHON patients without and with a history of significant recovery after disease onset, as defined by a 2-line increase on the Snellen chart (N = 6 out of 22 treated patients had a history of significant visual recovery). In contrast, Manners et al. (2015) found no relationship between diffusion parameters in post-geniculate visual structures and visual recovery.

In sum, MRI study results show that idebenone may help improve visual function by preventing widespread WM damage in the brain of LHON patients. However, a clear understanding of what mediates these effects and why they are variable and slow is still lacking.

### 3.2 Acute LHON

Few MRI studies have investigated changes in neural structure and function in the first year after initial LHON diagnosis. This time window, known as the acute phase, is marked by rapid degeneration of retinal ganglion cells leading to acute and severe loss of visual function.

In line with several case studies involving acute LHON patients (Batioǧlu et al., 2003, Honda et al., 2006; Lamirel et al., 2012), Blanc et al. (2018) reported T2 hyperintensities in the most proximal portion of the optic nerve and in the part extending to the optic chiasm of 28 acute LHON adults (Table 2). In T2-weighted images, hyperintensities may have several causes including axonal loss, demyelination, or trauma-related gliosis (growth of glial cells). Images consistent with swelling of the optic nerve and chiasm are often detected by neuroradiologists examining T2-weighted MRI images during the acute phase (Phillips et al., 2003, Ong et al., 2013, Mercuri et al., 2017; Lamirel et al., 2012; Blanc et al., 2018; Table 2). However, Blanc et al. (2018) did not find that mutation type related to any notable differences in signal brightness in the optic nerve and chiasm in the early (<3months) versus late (3–12 months) acute phase.

Clinical MRI is often performed with gadolinium, a paramagnetic contrast-enhancing agent administered via intra-vascular injection. It shortens the T1 time of the extracellular and extravascular spaces that it can reach, which is governed by the local physiology. Findings of enhanced gadolinium-induced contrast has led to speculation about inflammation in several case studies of acute LHON, potentially reflecting the disruption of the blood-brain barrier as a disease process or some type of auto-immune syndrome (Phillips et al., 2003, Vaphiades et al., 2003, Ong et al., 2013, Mercuri et al., 2017, Batioǧlu et al., 2003; Lamirel et al., 2012; Blanc et al., 2018). However, other studies, including the large-N study by Blanc et al. (2018), did not replicate this finding (Mashima et al., 1998, Honda et al., 2006, Thouin et al., 2013, Blanc et al., 2018). Thus, these inflammatory processes may not always be present at the time of the MRI evaluation.

Currently, most MRI studies targeting changes in the acute phase of LHON are qualitative single-case studies (Table 2), with abnormalities detected by expert neuroradiologists not blind to the diagnosis. To better understand the post-retinal neural mechanisms involved early on in LHON and their role in prognosis, objective, longitudinal quantitative MRI measures are needed. This is challenging however, as LHON is a rapidly progressing disease with a very short time window for investigating structural changes during the acute phase.

### 3.3 Unaffected LHON carriers (asymptomatic LHON)

Not all individuals carrying LHON-specific point mutations in their mitochondrial DNA develop the disease. These individuals are in the *asymptomatic* phase, and usually have good visual acuity, normal ocular examination, and no changes on the projection surface of the eye (i.e., fundus). Comparing asymptomatic individuals who do versus do not go on to develop LHON may provide important insights into pre-clinical markers that predict versus protect from this disease. To date, only a few neuroimaging studies have investigated changes in the brain of asymptomatic LHON individuals (Rizzo et al., 2012, d’Almeida et al., 2013, Mateus et al., 2016, Long et al., 2019; Table 2, Table 3, Table 4).

Whilst asymptomatic carriers do not suffer from clinically meaningful levels of visual impairment, they may present with subclinical reduction of chromatic sensitivity and visual field sensitivity, mostly within the paracentral ring (d’Almeida et al., 2013, Mateus et al., 2016, Long et al., 2019). Evidence for thickening of the most peripheral macular rings and retinal nerve fibre layer (RNFL), especially in the inferior and temporal quadrants, were also found in asymptomatic carriers (Savini et al., 2005, Mateus et al., 2016, Long et al., 2019, Carelli et al., 2019). This is in line with the swelling of the RNFL reported in the early stage of the disease (i.e., acute). However, whether this swelling during the acute phase is caused by similar processes as the swelling in asymptomatic LHON carriers is unclear (Savini et al., 2005, Barboni et al., 2005, Mateus et al., 2016, Long et al., 2019).

When investigating retinotopic visual cortex of 15 asymptomatic LHON carriers, all harbouring the m.G11778A variant, D’Almeida et al. (2013) reported increased cortical thickness in V2 and V3 of asymptomatic carriers compared to age-matched controls, whilst no difference in V1 thickness was found between groups (Table 4). These extrastriate changes appeared to be driven by visual area-specific differences between the younger and older asymptomatic carriers: the thickening of V2 relative to controls was most prominent in young asymptomatic LHON carriers (≤21yrs; N = 7), whilst the thickening of V3 was most evident in older asymptomatic carriers (>21yrs; N = 8). In a follow-up study by Mateus et al. (2016) involving in the same cohort of asymptomatic LHON carriers, greater cortical thickness was mainly observed in the periphery of V2 and V3, when compared to controls (Table 4). The authors suggested that these findings may reflect reorganisation of the cortex of asymptomatic LHON carriers, due to sub-clinical loss of paracentral visual field sensitivity and increased thickness of the most peripheral macular ring measured in these carriers (d’Almeida et al., 2013, Mateus et al., 2016; Table 4). Thus, notable effects of genetic variants may already be present at a cortical level in the asymptomatic stage when LHON genetic carriers do not present with clinically meaningful vision loss or scotoma. Moreover, longitudinal changes in the visual regions initially identified in these studies could offer new potential biomarkers of LHON that can be monitored to track the evolution of the disease. That said, what biological processes are responsible for the reported greater thickness of visual cortex in normally sighted asymptomatic LHON carriers is currently unclear.

When investigating water diffusivity in the brain of 11 asymptomatic LHON carriers using DTI, Rizzo et al. (2012) found no difference in mean diffusivity in optic radiations, prefrontal WM, or cerebellar WM compared to age- and sex-matched controls (Table 3, Table 4). Long et al. (2019) also observed no group differences in diffusion along the visual pathway from the retina to V1 in 14 asymptomatic LHON carriers compared to age-matched controls, but they did observe restricted diffusion (i.e., reduced FA and increased MD) in white-matter tracts connecting visual areas to more anterior brain regions (i.e., the bilateral inferior fronto-occipital fasciculi and bilateral inferior longitudinal fasciculi; Table 4). These changes in central white matter tracts mainly affected radial diffusivity, which has been linked to demyelination of the white-matter tracts, rather than axial diffusivity (Wheeler-Kingshott and Cercignani, 2009, Winklewski et al., 2018).

The lack of detectable reduced diffusivity in primary visual pathways of asymptomatic LHON carriers contrasts with studies in chronic patients with similar sample size (Milesi et al. 2012: N = 13; Manners et al., 2015: N = 17; Ogawa et al., 2014: N = 6; Takemura et al., 2019: N = 7), and suggests that this neural marker of LHON may predominantly develop around or after clinical onset of LHON. However, widespread changes in neural connectivity beyond the primary visual system, in DTI markers linked to demyelination, may indicate the existence of more widespread impacts on white matter, and potentially myelin integrity, in LHON gene carriers. These pre-clinical changes may be relevant to the co-morbidity of LHON with other demyelinating diseases such as multiple sclerosis. Moreover, understanding if and how these subclinical changes in neural physiology and function impact on the quality of life of asymptomatic LHON carriers, and their likelihood of developing clinical LHON, could be beneficial for patient care.

## 4 Conclusions

We have reviewed the insights gained from MRI studies into changes occurring in LHON beyond the retina, and summarised these in Fig. 1. In the introduction, we raised five questions about the post-retinal neural profile of LHON and in this concluding section we discuss the degree to which these questions have been answered.

**Q1&2: Which changes in neural function and structure occur beyond the retinal ganglion cell layer? What are the mechanistic causes of these neural changes, and which microstructural process might they reflect?**

*Pre-geniculate visual structures:* With the acute onset of LHON symptoms, reflected first in rapid central vision loss, many MRI studies have revealed swelling of the optic nerve (Phillips et al., 2003, Ong et al., 2013, Mercuri et al., 2017; Lamirel et al., 2012; Blanc et al., 2018; Table 2; Fig. 1A), which tends to subside after the acute phase (i.e., the first 12 months). A reduction of volume in the optic nerve and tract also occurs after onset of the acute phase, paired with decreased FA and increased MD. Together, this indicates less restricted diffusion along the primary optic projection from the eye to the LGN (Milesi et al., 2012, Wang et al., 2017; Table 2; Fig. 1A). At present it is unclear whether the loss in neural structure and diffusivity in the optic nerve and tract reflect axonal loss, swelling, or demyelination. The question of when most of this loss occurs in these regions also remains unanswered; studies reporting correlations between these measures and disease duration in chronic patients suggest that this is a gradually progressing process, but findings for optic nerve, chiasm, and tracts are somewhat conflicting (correlation: Inglese et al., 2001; versus no correlation: Barcella et al., 2010, Milesi et al., 2012, Wang et al., 2017, Jonak et al., 2020a).

*Geniculate and post-geniculate visual structures:* LHON not only affects the optic nerves, but also the central nervous system structures they project to (Fig. 1A). Reductions of volume have been measured in the LGN, optic radiations, and V1 of chronic patients (Barcella et al., 2010, Jonak et al., 2020a; Table 3, Table 4). These are paired with less restricted diffusion in these structures (Rizzo et al., 2012, Milesi et al., 2012, Ogawa et al., 2014, Manners et al., 2015, Takemura et al., 2019; Table 3). Again, it is unclear whether this primarily reflects demyelination or axonal loss, and findings conflict about whether these changes are progressive with disease duration, so it is not clear why and when they occur. However, despite showing no noticeable changes in DTI measures of diffusivity along the visual pathways, there is already a measurable increase in cortical thickness in the low-level visual cortex in carriers of the LHON gene variants who do not show symptoms (Rizzo et al., 2012, Long et al., 2019; Table 3, Table 4). This suggests that brain alterations may occur even before symptoms become clinically significant.

*Beyond the primary visual cortex:* Though deficits are primarily vision-related, changes/differences have been observed beyond early visual areas in LHON patients (Fig. 1B). Reductions of volume and less restricted diffusion have been found in subcortical areas (Jonak et al., 2020; Fig. 1), in structures involved in the auditory system (Manners et al., 2015, Long et al., 2019, Jonak et al., 2020a), and in frontal regions (Manners et al., 2015, Long et al., 2019). Increased volume has also been reported in the ventricles (Jonak et al., 2020b) and the hippocampus (Grochowski et al., 2021), and were correlated with disease duration and age. Together, these findings suggest potential cross-modal deficits in LHON and raise the question of more general neural pathological mechanisms. Indeed, LHON and MS are known comorbidities, with evidence of shared locations of brain lesions (Pfeffer et al., 2014, Inglese et al., 2001, Matthews et al., 2015). This co-occurrence of LHON with other demyelinating diseases may suggest shared mechanisms between the two diseases, in particular regarding the demyelination processes.

**Q3: How do changes in neural structure and function predict visual outcome?**

Less MRI research has focussed on function/structure relationships, so little is known about how neurological changes beyond the retina in LHON contribute to patient vision. However, several studies have demonstrated that neural markers of LHON have functional relevance. In non-expressing carriers, sub-clinical reductions in paracentral visual field sensitivity and RNFL thickness were linked to abnormal thickness in visual cortex that encodes the corresponding visual field locations (d’Almeida et al., 2013, Mateus et al., 2016), thus demonstrating early subclinical functional impact of LHON-linked mutations. Moreover, the fact that reductions of volume in post-geniculate visual structures correlate with RNFL thickness (Barcella et al., 2010, Jonak et al., 2020a; Table 3) suggests that post-geniculate dystrophies are linked to the loss of RGCs, either resulting from the same pathological process at RGC death, or from neuronal deterioration due to loss of visual inputs. Two studies in chronic patients also showed that optic nerve and tract diffusivity were less altered in individuals with better visual acuity outcomes after idebenone (Milesi et al., 2012; Wang at al., 2017).

In the post-geniculate pathway, structural integrity measures of the optic radiation could discriminate patients with significant visual recovery from those without (Rizzo et al., 2012). This suggests that some brain alterations may potentially be reversible after treatment or compensated by mechanisms that are yet to be fully explored. With current gene-therapy treatments already showing promising outcomes for visual recovery in LHON, it becomes essential to better understand how changes observed in MRI support these changes in visual function.

**Q4: What are the key challenges and limitations for MRI research on LHON?**

Conflicting findings are abundant in the MRI literature on LHON; for example, regarding whether disrupted diffusivity along the visual pathway is due to axonal loss, demyelination, or both (Ogawa et al., 2014, Takemura et al., 2019), and whether volume loss and changes in diffusion in pre-geniculate visual structures progressively worsen with disease duration (correlation: Inglese et al., 2001, Rizzo et al., 2012, versus no correlation: Barcella et al., 2010, Milesi et al., 2012, Wang et al., 2017, Manners et al., 2015, Jonak et al., 2020b, Jonak et al., 2020a). This in part stems from the inclusion of adults with chronic LHON presenting comorbidities with other demyelinating diseases such as multiple sclerosis (Inglese et al., 2001), potential treatment with idebenone (Rizzo et al., 2012), and the genetic variability of the disease. As all these factors affect both prognosis and recovery, they are likely to present with different underlying neural profiles.

MRI findings of LHON are also limited because the disease is relatively rare, and the onset and progression to near-blindness is very rapid. As result, most studies have a small sample size and patients are usually only recruited in the chronic phase of LHON. This limits the generalisability of the results and the power to detect subtle differences in neural disease markers. As a result, we have little understanding of how early-stage disease progresses over time in visual brain regions beyond the retina. With the development of regenerative treatments such as gene therapies (see response to Q5 below), insight in these early processes and how they may interact with treatment will become crucial in coming years. Further work would benefit from larger sample sizes as well as recruiting patients in various stages of the disease. However, the fast progression of the disease represents a challenge, and the latest and most informative functional and structural MRI measures of the visual brain may not be readily available in all places. New advances in quantitative MRI technology may help address this issue (see response to Q5 below).

A final challenge of this research is that imaging of visual brain structures can be particularly difficult due to their tissue properties. For example, comparisons of MRI measures of the optic nerve and other visual structures is complicated by the fact that the optic nerves are particularly difficult to image due to their small size, and their proximity to orbital cavities (Hoch et al., 2017). The latter can cause signal dropout and severe eye-movement-related image artifacts. Imaging this structure typically requires a restricted volume to achieve sufficient resolution in clinically feasible acquisition times. As a result, MRI sequences are differentially optimised to visualise either the optic nerves or tracts and other structures, meaning that they are usually investigated in separate studies and patients. This makes it hard to assess progression of the disease along the visual pathway in the same patient.

**Q5: What are the most important open questions about LHON that MRI could help resolve?**

Recently, novel imaging methodologies have been developed that have the potential to provide more direct ways to assess microstructural changes in vivo. For instance, quantitative MRI measures can be used in biophysical modelling to calculate metrics that directly characterise biological properties of the tissue, such as the g-ratio, which quantifies the ratio of axonal diameter with and without the myelin sheath (Mohammadi & Callaghan, 2021). This estimate has the potential to differentiate demyelination (reduced g-ratio) from inflammation (unchanged g-ratio). When combined with longitudinal studies, this could help disentangle contributions of demyelination, inflammation, and axonal loss to the LHON profile. Moreover, these quantitative measures are scanner-independent. This provides exciting scope for improving sample sizes in this field by permitting comparable data collection across multiple specialist centres, and by increasing the reliability of longitudinal research in individual patients.

Though neuroimaging studies have provided insight into how LHON impacts beyond the retina during the disease, our understanding of the neural mechanisms supporting visual recovery is still in its infancy. For example, it is presently unclear what causes the spontaneous functional recovery in a small but substantial percentage (<20 %) of patients with LHON, and in particular those with the T14484C variant. To date, no neuroimaging studies have investigated spontaneous recovery in LHON, nor any differences in neural structure and function between genotypes that may facilitate this. Potential explanations for this recovery include the reviving of deactivated but viable RGCs (Acaroğlu et al., 2001, Sharkawi et al., 2012), re-myelination of axons, or functional compensation processes at the level of visual cortex or LGN (d’Almeida et al., 2013, Rocca et al., 2011). Such functional compensation may, for example, involve increased allocation of neural resources to spared retinal regions (Baseler et al., 2002, Baker et al., 2005, Castaldi et al., 2020) or altered attentional processes (Dumoulin & Knapen, 2018), as observed in other types of visual field loss. Whilst important, investigating spontaneous recovery in LHON is challenging as it is unpredictable and thus would require regular measurement.

Another important future direction will be to gain better insight into the early dynamics of visual pathway structure and function loss in LHON. This will be valuable in defining a treatment window for gene therapy and evaluating treatment impact. Recent approaches work by injecting patients with an adeno-associated virus (AAV2/2-ND4) that safely replaces affected mtDNA in the mitochondrial complex I, to prevent loss of RGCs (Koilkonda et al., 2014, Wan et al., 2016, Newman et al., 2021). Two recent clinical trials that use this approach revealed significant improvements of visual acuity and contrast sensitivity in the treated eye. After 2 years, >70 % of LHON patients’ acuity measures improved, to an equivalent of 0.3logMAR, in their treated eye (Newman et al., 2021, Wan et al., 2016, Yu-Wai-Man et al., 2020, Moster et al., 2020). Surprisingly, however, similarly improved acuity after 2 years was also observed in the non-injected eye, complicating the interpretation of these results (Yu-Wai-Man et al., 2020). It is likely, however, that this change is pertinent to treatment, as a recent large study which compared an indirect LHON control group to the change observed after gene therapies, showed a progressive improvement of visual acuity from 12 to 52 months in the treated group compared to the external control group, which became significant after 18 months and clinically significant after 48 months. This bilateral benefit raises questions about whether a transfer effect is operational at the viral level only or also involves neural plasticity. Functional MRI could help address these questions by measuring how and when neural visual function for input from both eyes changes after gene therapy.

MRI could be a powerful technique capable of sensitively detecting the recovery of function and structural integrity in response to treatment. The value of fMRI as an outcome measure for gene therapy has been demonstrated in Leber’s Congenital Amaurosis (LCA), a condition associated with severe blindness from birth accompanied by nystagmus and light sensitivity (Ashtari et al., 2011, Ashtari et al., 2015). Ashtari et al. found that after gene therapy in 3 LCA patients, cortical responses in visual areas were higher for high- and medium contrast checkerboard patterns presented to the treated compared to the untreated eye, whilst behavioural assessments showed only modest improvement. The distribution of cortical activation also correlated partially with visual field maps measured behaviourally. These results suggest that visual function after gene therapy is restored, with potential enhancement of contrast sensitivity, at least at the cortical input stage. Functional MRI appeared to be sensitive to treatment-mediated changes in the brain well before the emergence of behavioural improvements. Similar benefits of fMRI for gene therapy assessment have also recently been demonstrated for achromatopsia, a congenital disease that disrupts the function of cone photoreceptors. In 2 out of 4 treated patients, fMRI measures revealed that when cone photoreceptors in the retina were stimulated no retinotopically organised response was measurable in primary visual cortex before treatment. After treatment, a clear response emerged that was paired with an improvement in perception (Farahbakhsh et al., 2020).

**Final Summary**

In sum, MRI has provided important insights into the neural aetiology of LHON and its wider impacts beyond the optic nerve, as well as into the potential neural mechanisms of treatment effects. However, many questions remain to be resolved, including which processes explain these changes at the microstructural level and their commonality with other neurological diseases, the timing of these changes and how they relate to visual function, and the causes of recovery observed in some cases. To address these important questions and pave the way for future MRI applications that can inform emerging new treatments of LHON, it will be crucial to collect data from larger groups of patients, separate out neuropathology for different phenotypes, improve ways of measuring visual function at the brain level in LHON, and utilise recent quantitative MRI approaches to distinguish between candidate recovery mechanisms such as remyelination of affected CNS fibre tracts or restoration of central vision function.

## Declaration of Competing Interest

The authors declare that they have no known competing financial interests or personal relationships that could have appeared to influence the work reported in this paper.
